# The mental health disaster in conflict settings: Can scientific research help?

**DOI:** 10.1186/1471-2458-7-275

**Published:** 2007-10-02

**Authors:** Frank Neuner, Thomas Elbert

**Affiliations:** 1 Dept Psychology, University of Konstanz, Germany

In the current issue of *BMC Public Health*, Asma Al-Jawadi and Shatha Abdul-Rhman have published a remarkable study [1]. They assessed 3079 children from families who attended primary health care institutions in Mosul, Iraq and found mental disorders in more than one third of the children. This is further proof that children in today's conflict regions are severely affected by war.

These results fit with a substantial body of epidemiological research, which has consistently shown that mental disorders become common in populations affected by war and conflict. The most prevalent set of symptoms corresponds well with posttraumatic stress disorder (PTSD). Endemic rates of PTSD, affecting at least every sixth person and sometimes even every other person, have been found among adults [2-5] and children [6,7] alike. Given that a mental disorder implicates significant functional impairment, and given that nearly half of countries worldwide harbour current conflict zones or bore witness to a recent war [8], these figures are shocking. PTSD rates continue to rise, unsurprising given that systematic atrocities, massacres, and attacks are often applied as deliberate strategies [9] to inflict suffering. Indeed, the likelihood of developing PTSD depends on the cumulative amount of traumatic stress experienced [10]. With its substantial sample, the Asma Al-Jawadi and Shatha Abdul-Rhman study not only validates this view, but the results show that many children are obviously severely deprived. The real figures for psychological dysfunction in Iraq may be even higher for two reasons: first, the poorest strata of the population was probably neglected in the sampling procedure and second, because the researchers did not use a standardized instrument to screen for the range of symptoms in the individuals. As a result, these data probably lead to an underestimate of the real prevalence.

Evidence of the mental health disaster, accentuated by Asma Al-Jawadi and Shatha Abdul-Rhman has been accumulating for more than two decades [11]. With the range of symptoms and disorders observed in children, the Mosul study adds two new frightening dimensions to the catastrophe: (1) a combination of stressors, including traumatic stresses, but also poverty, unsafe living and poor nutrition comprises an unhealthy mixture that prevents normal development in a significant proportion of the children (2) the observation that children are inhibited from normal development leads to the prediction that society at large will become even less functional in the future.

What gains have been made in the fight against traumatic disorders and other mental health problems in conflict areas? What do we know about the impact on individual, family and community functioning? Given what we know about the effects of trauma, it is likely that we will also see a rise in substance abuse and suicidality [12], violence [13], and a worsening of physical health [14]. Mental illness reduces psychological functioning on all levels. Consequently, a major impact on the economic development of the war-affected region, as well as on the continuation of conflict is to be expected. But to date there has been no research testing this statement. The intervention programs that have been implemented are seldom evidence-based and have not been tested for their efficacy. One might wonder what the respective WHO branches have been doing for the last 20 years. Funding agencies consider it obligatory to support science when investigating cycles of the malaria parasite, yet will balk at the idea of investigating cycles of violence. NGOs have started to offer what they refer to as psychosocial assistance in crisis regions. But what treatment would the expert order when there is no diagnosis and no set of scientifically sound intervention trials? Treatment protocols are being developed *ad hoc *by untrained individuals and passed off as acceptable when used on indigenous people. It seems there are few randomized trials on the treatment of trauma-related disorders in conflict zones [15]. In other words, there is little intervention research for people living in traumatized regions.

Why has progress been so limited in this area? There seem to be three major obstacles: (1) the donor (2) the reviewer and (3) the expatriate:

(1) There is a lack of reward when it comes to solving the problems of the poor and those living in less developed nations. The situation for these populations is not much better for parasitic diseases that affect mainly the poor, such as sleeping sickness. The Western world does not benefit from research in this area and in science it is considered exotic, an "Orchideenfach" (orchids are beautiful but bear no fruits).

(2) Reviewers often argue that rigorous scientific standards cannot be met in developing countries or conflict regions. There are too few qualified researchers to collaborate with. There are no reliable sources to be used for proper sampling, no telephones available for random-digit dialing. Complicating matters is the fact that in one region multiple languages may be spoken. Validated instruments for measuring variables are nonexistent. There is no proper accommodation for foreign scientists, some areas are difficult to access, and quite often even in peaceful regions, there remains a constant threat of insurgency. Migration hampers the longitudinal observation of subjects, and travelling in these regions can be stressful and dangerous.

Asma Al-Jawadi and Shatha Abdul-Rhman have demonstrated that high quality research is possible, even in countries suffering from war. Of course, the methods could have been improved, and with some creativity and funds most of the critics could have been silenced, even though we doubt that the outcome would have changed qualitatively. From a scientific perspective, hut-to-hut sampling is far better than random-digit dialing. At present, there is no major funding agency that would support sound epidemiological research of mental health in Iraq. It is as if mental health is not considered to be an emergency. We need more studies like the present one, that show that the work can be done and we need grant reviewers that realise that renting a vehicle in countries like Iraq is expensive but who also know that the payment for an excellent work force is inexpensive, allowing for innovative designs.

(3) We have worked in conflict zones such as Afghanistan, Rwanda, Sri Lanka, Somalia or Uganda and we continue to hear the same arguments from expatriates and consultants of various NGOs: high standard quantitative empirical research may well be possible, but the cultural and social context in conflict areas leaves any such "culturally inadequate" attempt undesired. It is intrusive and even "immoral". The transfer of "Western" concepts and techniques to war-affected societies in developing countries risks "perpetuating the colonial status of the non-western mind" as every "culture has its own frameworks for mental health, and norms for help-seeking at times of crisis" [16].

This reasoning assumes a clear distinction between Western "Eurocentric" cultures and other cultures in non-industrialized countries. Such a distinction seems to be straightforward, but is it valid? The concept of homogeneous cultures is neither true for industrialized nor for developing countries. In all regions there exists a wide diversity of attitudes, values and habits, and neither artificial borders set between countries (or between the industrialized and non-industrialized world) can serve to demarcate differing cultural values. The same is true for health and mental concepts: many societies in developing countries have already chosen to adapt mental health concepts developed by Western psychiatry and prefer the corresponding treatment methods rather than "traditional healing". At the same time, particularly in rural areas in Europe traditional healing techniques for physical and mental complaints have remained popular. Those who emphasize the differences between cultures and advocate non-interference in cultures that are considered to be "traditional" use an ethical argument. As a matter of political correctness, terms such as "culturally sensitive" are now commonly included in mental health proposals and articles. It is not straightforward to favour the position of not interfering in cultural traditions, norms and beliefs in psychosocial work. All cultures are constantly changing and the idea that there are any cultures that fully rely on traditional norms and have not been affected by the modern world is a romantic view. The consequences of not interfering in cultural norms would also include withholding knowledge about scientific methods of objective assessment and evaluation from these cultures. It is these methods that have led to the development of treatment approaches that have proven to be effective and to the identification of less successful or even harmful methods. More generally, the same research methods have led to the industrialization and the currently dominating positions of the industrialized countries. Withholding this knowledge and science as a powerful tool from developing countries might slow impact on traditional cultural norms, yet at the same time aggravate the global discrepancies in development and power. Protecting societies that are considered to be traditional from modern progress risks building cultural reservations of societies that remain dependent on the goodwill of the powerful.

Ironically, arguments about the cultural difference between countries has been primarily raised by European or North American "experts" with the intention of protecting the poor in the developing countries from the West, or more precisely from its educated scientists and clinicians. Upon closer examination this appears to be a colonial perspective. Like many other professionals from crisis regions, Asma Al-Jawadi and Shatha Abdul-Rhman are pragmatic enough to build on concepts that have been developed in Western science. It is note- and praiseworthy that researchers from Iraq base their study on the latest science, regardless of whether the mental health manual is sanctioned by the United States. Mental health professionals from resource-poor and conflict zones of this world are open to see what they chose, open to cooperate, to teach and to learn. So now it is up to donors, funding agencies and internationally leading researchers alike to take the opportunity to participate in this exciting field of research. We are living in a period of globalisation, not only economically but also in terms of violence, terror and conflict. Eventually the view will prevail that scientists should be permitted and encouraged to conduct this vital research. While we wait for this cultural shift in conflict research, a tangible reward we have is the look of relief in the eyes of children who have been helped to stop the mental war raging in their minds.

## Pre-publication history

The pre-publication history for this paper can be accessed here:


